# Texture analysis of protein deposits produced by droplet evaporation

**DOI:** 10.1038/s41598-018-27959-0

**Published:** 2018-06-25

**Authors:** Yojana J. P. Carreón, Maricarmen Ríos-Ramírez, R. E. Moctezuma, Jorge González-Gutiérrez

**Affiliations:** 1 CINVESTAV-Monterrey, Apodaca, 66600 Mexico; 2 CONACYT-Instituto de Física “Manuel Sandoval Vallarta”, San Luis Potosí, 78000 Mexico; 3 Departamento de Termofluidos, Facultad de Ingeniería, Universidad Nacional Autónoma de México, Av. Universidad 3000, Mexico, D.F. 04510 Mexico

## Abstract

The deposit patterns derived from droplet evaporation allow current development of medical tests and new strategies for diagnostic in patients. For such purpose, the development and implementation of algorithms capable of characterizing and differentiating deposits are crucial elements. We report the study of deposit patterns formed by the droplet evaporation of binary mixtures of proteins containing NaCl. Optical microscopy reveals aggregates such as tip arrow-shaped, dendritic and semi-rosette patterns, needle-like and scalloped lines structures, as well as star-like and prism-shaped salt crystals. We use the first-order statistics (FOS) and gray level co-occurrence matrix (GLCM) to characterize the complex texture of deposit patterns. Three significant findings arise from this analysis: first, the FOS and GLCM parameters structurally characterize protein deposits. Secondly, they conform to simple exponential laws that change as a function of the NaCl concentration. Finally, the parameters are capable of revealing the different structural changes that occur during the droplet evaporation.

## Introduction

The patterns formed by the evaporation of droplets are in the deposition of materials, inkjet printing^1–3^, DNA/RNA recognition^4–7^ amoung many other systems. In the context of the diagnostic of pathologies, the structural characteristics of deposits enable detection of leukemia, anemia, tuberculosis, cancer, among many other health problems^8–10^. The final morphology of a deposit depends on transport mechanisms and aggregation processes of colloidal particles. The competition between capillary flows driven by continuity and Marangoni flows driven by surface tension gradients, determine the mass transport in the interior of a droplet^11^. The capillary flows move radially outwards to compensate the loss of mass due to the evaporation of water molecules occurs mostly from the edge of the droplet. Under this process, the particle deposition occurs at the contact line, forming the so-called “coffee ring”^12–14^. Marangoni flows are generated by surfactants and temperature gradients that force the fluid to circulate inwards of the droplet. Indeed, this transport mechanisms can eradicate the coffee ring formation^15–18^. On the other hand, the aggregation phenomenon is mediated by forces (friction *F*_*drag*_, the electrostatic *F*_*ex*_ caused by the charges of the molecules, and adhesion *F*_*ad*_, between the macromolecules and the glass substrate) that emerge during the evaporation process^2,14,19–25^.

The study of protein solutions is a good starting point for understanding the transport mechanisms and aggregation processes during droplet evaporation of biofluids. The protein-salt interaction allow the formation of a large variety of complex aggregates^26–30^. Deposits composed by lysozyme and NaCl exhibit an amorphous peripheral ring and dendritic shapes^31,32^, while protein deposits of BSA and NaCl show structures such as rosettes, scallops, Chinese arrows and zigzag patterns^28^. Indeed, different salts interacting with the same protein generate different aggregates^33^. These structures arise from complex mechanisms of aggregation of ions on a protein film^29^. On the other hand, recently we scrutinize on the pattern formation produced by the droplet evaporation of suspensions containing two different proteins^34^. We found that salt is unnecessary for the formation of crystal clusters, dendritic and undulated branches, and interlocked chains inside of a small region of the deposits. These structures appear in a small region inside of deposits.

The standard procedures for the morphology assessment of deposits are carried out using the radial density profile and the normalized grayscale intensity profile. These quantities correlate the morphological aspects of deposit patterns with components in an aqueous solution and the state of such components^18,35–39^. The radial density profile statistically describes the whole mass distribution as a function of the distance to a given point, usually from the center to the edge of the deposit; while the line profiles give information on the distribution of mass on a narrow segment. The ability of these quantities to capture and differentiate the geometry of aggregates shows some limitations. For example, a small curve in the radial density profile could be correlated to a cluster of small aggregates, or only to a large aggregate. Therefore, although refined strategies have been implemented to capture some specific characteristics of deposits^33^, there is still a need to implement statistical parameters capable of characterizing and differentiating structural aspects of deposits.

In this paper, we report the study of the structure of deposit patterns formed by the evaporation of droplets of protein mixtures containing NaCl. Optical microscopy reveals an enormous diversity of complex patterns that emerge at a different relative humidity of the evaporation environment (RH), salt concentration, and relative protein concentration. We use the first-order statistics (FOS) and the gray level co-occurrence matrix (GLCM) statistics as a natural strategy to fully capture the complex texture of deposit patterns. The FOS and GLCM are complementary measures to the assessment of the texture of an object. They are used to characterized morphological changes in tissue collagen fibril organization caused by pathological conditions^40^, cancer classification^41,42^, among many other systems of relevance^43,44^. FOS textures depend on the gray tone distribution of pixels intensity of the image of an object, while GLCM (second-order statistics) is related with the spatial arrangements of pixels intensities in a region of interest. In other words, GLCM parameters are calculated from a matrix based on the interpixel correlation of the image^45^, while FOS neglects pixel relationships. The main attraction of GLCM is that these parameters act as indexes on the frequency of combinations of gray levels in an image. We found that these texture parameters collapse to simple exponential laws that change as a function of the NaCl concentration. Furthermore, the combination of the FOS and GLCM statistics allows classifying deposits at different relative protein concentration with an accuracy above 95%. Finally, the texture analysis reveals three sequential stages of aggregation: coffee ring formation, the crystals growth, and water drying stage.

## Results

### The effect of the NaCl concentration on the texture of deposits

**Figure 1:**
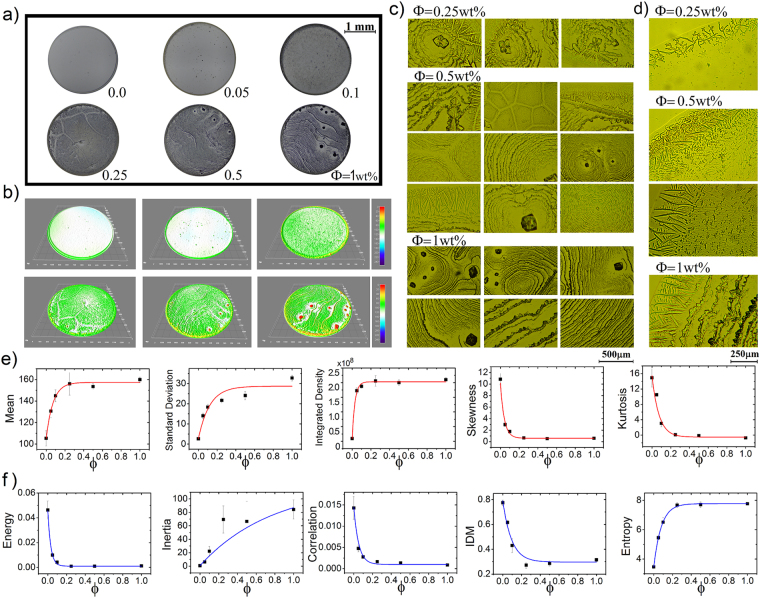
Protein mixtures deposits formed at different NaCl concentration. (**a**) Deposits formed during the evaporation of droplets containing two types of proteins (relative concentration *ϕ*_*r*_ = 1:1 and *ϕ*_*p*_ = 0.1 wt%) at NaCl concentration *ϕ* = 0, 0.05, 0.1, 0.25, 0.5, and 1 wt% and T = 37 °C. (**b**) The corresponding three-dimensional light intensity profiles. Patterns formed at the center of the deposits (**c**) and the edge (**d**). Texture analysis of deposits formed at different NaCl concentration. The corresponding texture parameters (**e**) FOS (Mean, Standard Deviation, Integrated density, Skewness and Kurtosis) and (**f**) GLCM (Energy, Inertia, Correlation, IDM and Entropy). The blue and red lines are the best fit for the curves. The error bars correspond to standard deviations from *n* = 24.

We first explore the effect of the NaCl concentration on the pattern formation of protein deposits. Figure 1a shows six deposits generated by a mixture of BSA and Lysozyme (relative protein concentration *ϕ*_*r*_ = 1:1 and protein concentration *ϕ*_*p*_ = 0.1 wt%) at different NaCl concentration (*ϕ* = 0, 0.05, 0.1, 0.25, 0.5, and 1 wt%). At first glance, the salt concentration increases the structural complexity of deposits. The three-dimensional representations of such deposit images reveal that salt induces the formation of crystals of different size and shapes, see Fig. 1b. Figure 1c shows that deposits formed at *ϕ* = 0.25 wt% contain star-like salt crystals surrounded by a semi-rosette pattern assembled by needle-like and scalloped lines structures. For *ϕ* = 0.5 and 1.0 wt% it is observed a more saturated rosette-shaped protein pattern which is associated with an increase in prism-shaped stacks crystals. Small interconnected tip arrow-shapes sculpt scalloped concentric rings that compose the groups of rosettes. For *ϕ* = 1.0% the formation of rosette structures is interrupted by crystals of prism-shaped stacks. Finally, the edge of *ϕ* = 0.25 wt% deposits contain a dendritic pattern, while for *ϕ* = 0.5 and 1.0 wt% result in needle-like or interconnected needle-like crystals, see Fig. 1d.

We carry out the FOS and GLCM statistic on the deposits in order to characterize the effect of the NaCl concentrations on the structural changes in the protein deposits. Interestingly, the FOS and GLCM parameters change exponentially as the NaCl concentration as follows:

1 $$\delta =-\,{\delta }_{0}{e}^{(-\varphi /k)}+{\delta }_{s},$$

where *δ*_0_ is the texture parameter value of the surface, *δ*_*s*_ is a value where the texture parameters *δ* saturates, and *k* is the characteristic concentration *ϕ*_*c*_ at which the magnitude of *δ*_0_ diminishes by a 1/*e* factor. The FOS texture parameters extracted from the deposits formed at different NaCl concentration are plotted in Fig. 1e. The Mean, Standard Deviation, and Integrated Density parameters grow due to crystals increase the roughness on the deposits surface and the pixels intensity of the image. On the contrary, the Skewness and Kurtosis decrease because the gray level intensity distribution of the histograms presents a more asymmetrical pattern right-tailed at high NaCl concentrations. Figure 1f shows the behaviour of the GLCM parameters calculated from the texture analysis of the deposits. The Energy, Correlation, and IDM parameters decrease exponentially due to the reduction of the textural uniformity, the similarity on gray-level regions, and the local homogeneity in the deposits, respectively. On the other hand, the Inertia and the Entropy increase exponentially by increasing the number of pixels in large contrast as well as heterogeneous regions in an image.

Image analysis has less consistent results to differentiate among deposits formed at high NaCl concentrations. This is probably because of the high number of crystals of different shapes which saturate the difference in the frequency and the intensity of the values of the grey levels of each pixel of the image. Indeed, these crystals diminish the influence of the large aggregates that appear covering small regions in the deposits formed at 1.0 wt%. We believe that this behavior reflects a threshold for the saturation of aggregates on protein deposits.

### The effect of the relative protein concentration on the texture of deposits

**Figure 2:**
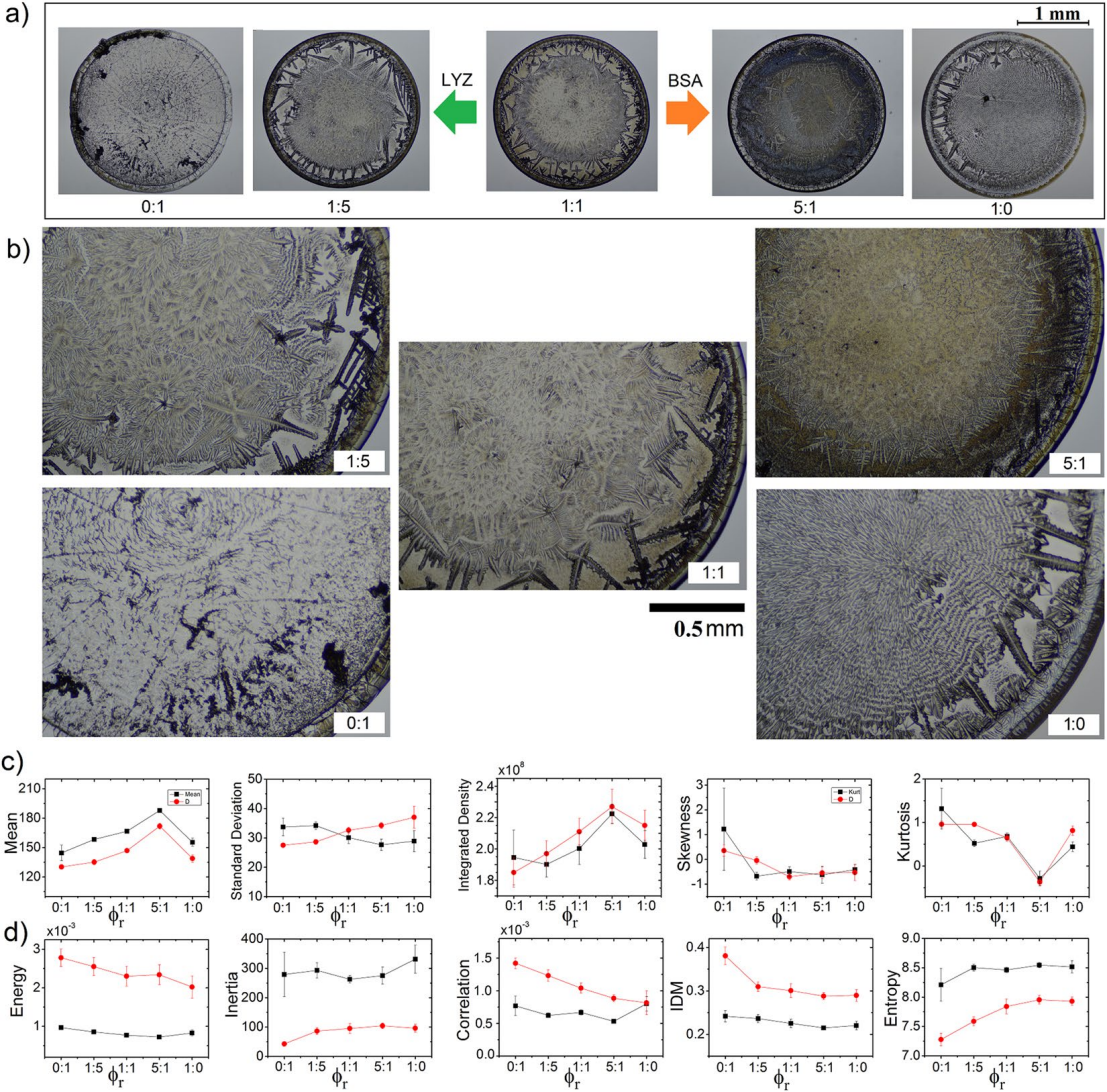
Protein mixtures deposits formed at different relative concentration. (**a**) Deposits containing proteins mixture of BSA-LYZ. Relative protein concentration *ϕ*_*r*_ = 0:1, 1:5, 1:1, 5:1, 1:0, protein contraction *ϕ*_*p*_ = 1.6 wt%, and NaCl concentration *ϕ* = 1 wt%. The droplet evaporation was carry out on a solid surface at T = 37 °C. (**b**) Zoom at representative patterns in the deposits. The corresponding texture analysis (**c**) FOS (Mean, Standard Deviation, Integrated density, Skewness and Kurtosis) and (**d**) GLCM (Energy, Inertia, Correlation, IDM and Entropy). Black squares and red circles correspond to proteins deposits at *ϕ* = 0.5 and 1 wt%, respectively). The error bars correspond to standard deviations from *n* = 24.

Next, we explore the effect of the relative protein concentration on the morphology of protein deposits. Figure 2a shows deposits at a different relative protein concentration *ϕ*_*r*_ = 0:1, 1:51:1, 5:1, 1:0 (protein contraction *ϕ*_*p*_ = 1.6 wt% and NaCl concentration *ϕ* = 1 wt%). All deposits show similar structural aspects. Regardless of the relative concentration, in protein deposits appear a coffee ring that interlocks to small crystals of a different shape. Indeed, for *ϕr* = 1:5, and 1:1, it is observed a small gap surrounding many interlocked crystals of different morphologies. However, a vast diversity of different characteristics appear in the interior of these deposits. Lysozyme deposits (0:1) shows heterogeneous patterns with dispersed amorphous crystals, in contrast to BSA deposits (1:0) that contain a big rosette pattern without a crystal core and needle-like structures, see Fig. 2b. For intermediate concentrations, *ϕ*_*r*_ = 1:5, 1:1, and 5:1, inner structures resemble dendritic shapes. We must remark that deposits formed at *ϕ* = 0.5 wt% show similar morphologies (see Fig. 1s in the supplementary material).

Figure 2c shows the FOS and GLCM values related to deposits generated at different relative concentrations of proteins *ϕ*_*r*_. Deposits formed at *ϕ*_*r*_ = 5:1 and *ϕ* = 1 wt% show the highest values in Mean and Integrated density; and lower values in Skewness and Kurtosis than the rest of the deposits. This occurs because they are assembled by a high number of crystals that fill the whole surface. Interestingly, deposits formed at *ϕ*_*r*_ = 0:1 shows the highest values of Standard Deviation. They are formed by crystals of a different size that promote lowest values in Mean and Integrated density; and most significant values in Skewness and Kurtosis. Figure 2d shows the corresponding GLCM parameters extracted from the deposits at a different relative protein concentration. Deposits formed at *ϕ*_*r*_ = 0:1 show the highest values in Energy, Correlation, and IDM. On the contrary, deposits formed at *ϕ*_*r*_ = 5:1 show the lowest values in these three parameters. Finally, deposits formed at *ϕ*_*r*_ = 0:1 reach the lowest values in Entropy, while deposits formed at *ϕ*_*r*_ = 1:0 show the highest values in Inertia. Note that excluding the standard deviation, the rest of the parameters corresponding to deposits formed at *ϕ* = 0.5 wt% show similar behaviors as a function of the relative protein concentration.

In order to scrutinize the ability of FOS and GLCM statistics to classify deposits that at first glance are structurally very similar, we perform canonical discriminant analysis to determine whether the complete sets (or separately) of FOS and GLCM parameters are useful in the deposit classification. This analysis generated canonical discriminant functions, which are linear combinations of the canonical variables (FOS and GLCM parameters) that maximized and minimized the variability among and within deposits groups, respectively. From the complete sets of FOS and GLCM parameters, the FOS parameters, and the GLCM parameters; the discriminant analysis give two, fourth, and one canonical discriminant functions, respectively. Table 1 in the supplementary material shows the unstandardized canonical coefficients corresponding to the canonical discriminant functions obtained from the discriminant analysis using GLCM. Figure 2s in the supplementary material shows the scatter diagram of the first two discriminant functions from the GLCM parameters. The five categories are separated clearly and that the members of each group are around the group centroid.

To characterize the discriminant ability of the canonical function is used the Wilks’ lambda. If the value of Wilks’ lambda is large, the means of groups are almost the same. If the value of Wilks’ lambda is small, there are differences between groups. When the means of groups are not equal, conducting discriminant analysis makes sense. Table 2 in the supplementary material shows small Wilks’ lambda values related to the above discriminant functions. Indeed, the table also provides small Chi-Square values that test the significance of Wilks’ Lambda; and provides *p*-values lower than 0.05 that conclude that the discriminant functions grouped into a proper category a given deposit. Finally, from the Error Rate table for training data in Table 3 in the supplementary material, we can conclude that the accuracy in the classification of the groups of deposits formed at *ϕ* = 1 wt% is 95% and 93.33% when only the FOS or GLCM parameters are used, respectively. However, the accuracy increases when both sets of parameters are used simultaneously.

### The effect of the relative humidity on the texture of deposits

**Figure 3:**
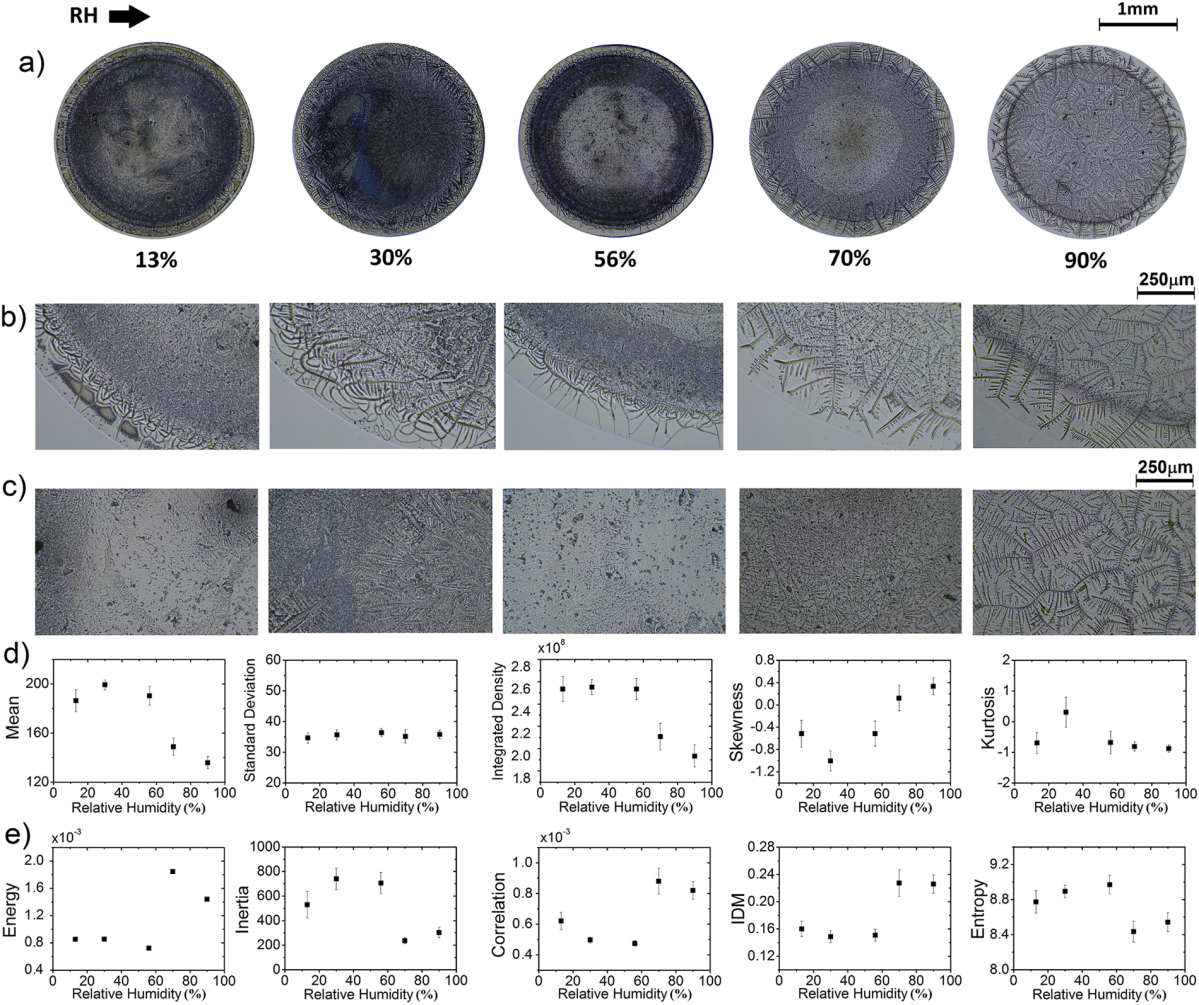
Proteins mixtures deposits formed at different RH. (**a**) Deposits containing protein mixture of BSA-LYZ and NaCl (protein concentration *ϕ*_*r*_ = 5:1, *ϕ*_*p*_ = 1.6 wt%, and NaCl concentration *ϕ* = 1 wt%) at different RH and T = 25 °C. Patterns formed at the edge (**b**) and center (**c**) of the deposits. The corresponding texture analysis (**d**) FOS (Mean, Standard Deviation, Integrated density, Skewness and Kurtosis) and (**e**) GLCM (Energy, Inertia, Correlation, IDM and Entropy). The error bars correspond to standard deviations from *n* = 24.

Some recent studies prove that the relative humidity (RH) affects the mass transport mechanisms and aggregation processes of particles during the evaporation of a droplet. In general, an increase in RH result in lower contact angle, more spreading of the droplet, and a decrease in the evaporation rate, thereby leads to a more substantial particle deposition area^46,47^. We explore the effect of relative humidity (RH) on the formation of pattern deposits with the aim to investigate the effectivity of the FOS and GLCM analysis to characterize structures. Figure 3a shows deposits formed at different relative humidity (protein concentration *ϕ*_*r*_ = 5:1, *ϕ*_*p*_ = 1.6 wt%, and NaCl concentration *ϕ* = 1 wt%). Two groups of patterns appear as a function of the RH. For lower RH (13–56%) it is predominantly observed in amorphous aggregates while at high RH (70–90%) deposits show crystallization in branched arborescent patterns. Figure 3b shows that in the peripheral region of the first group are located amorphous interconnected lines, dendrite shapes and radial cracks at the coffee ring zone. In contrast, the second group contains needle-like patterns. Figure 3c shows the inner region of such deposits. For RH of 13, 30 and 56% it is observed dispersed amorphous aggregates, while at high RH values exhibit branched arborescent formations that become larger as the RH increase.

Figure 3d,e show the FOS and GLCM values related to deposits generated at different relative humidity. Interestingly, results indicate that the deposit group formed at low RH (13–56%) show high heterogeneity and roughness. The global homogeneity and higher degree of similarity coexist in the deposits created at high RH (above 70%). Observing the values in Fig. 3d,e, it is possible to assess the ability of each variable in providing the characterization and distinction among deposits. Excluding the standard deviation, skewness, and kurtosis the rest of the parameter can distinguish between deposits composed of complex aggregates (RH = 13, 30 and 56%) and stains containing crystallization in branched arborescent patterns (RH = 70 and 90%).

### The evolution of the texture during the pattern formation

**Figure 4:**
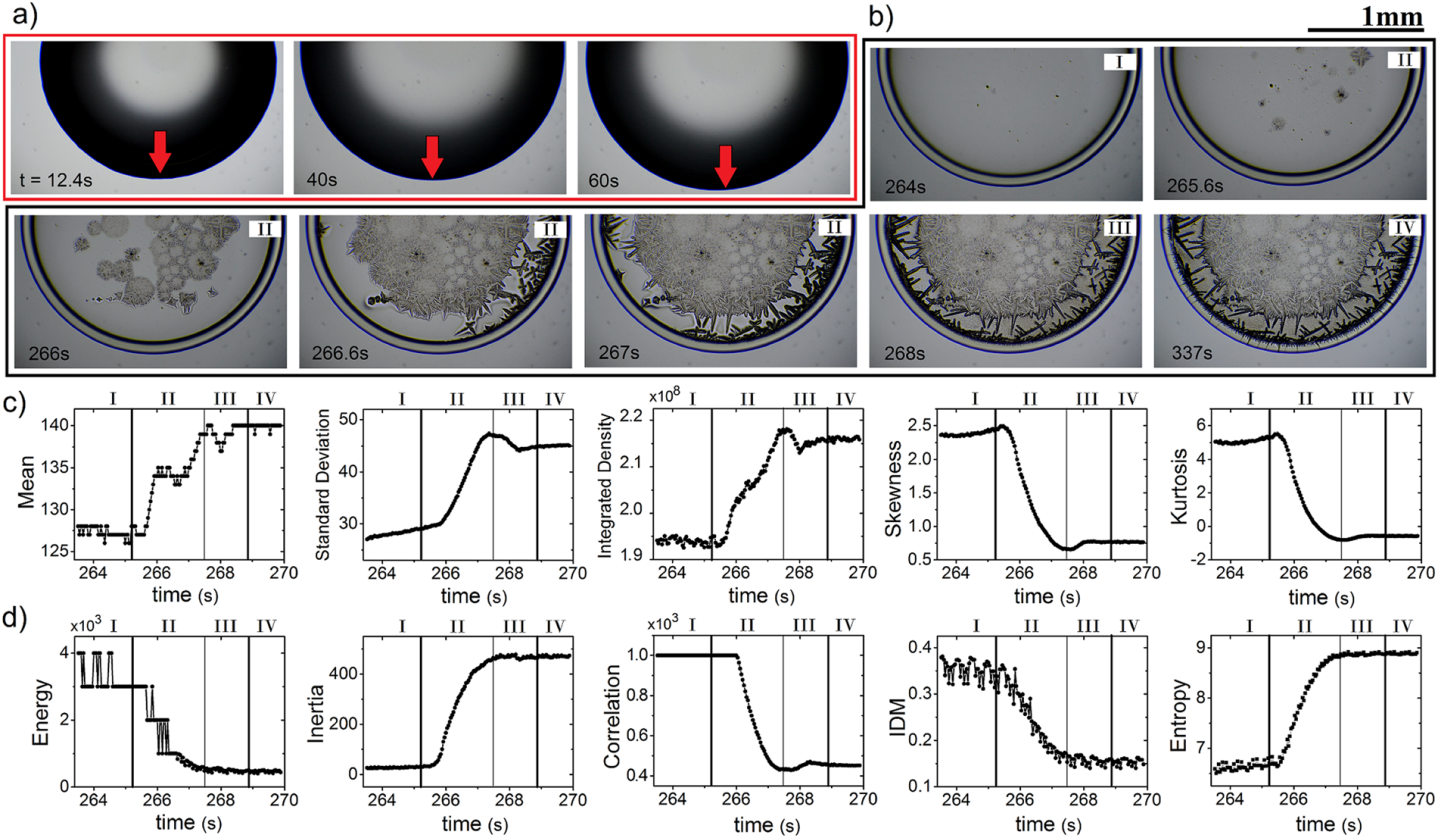
Texture analysis during the formation of deposits. (**a**) Sequential images of the expansion process of a BSA-LYZ droplet (protein concentration *ϕ*_*r*_ = 1:1, *ϕ*_*p*_ = 1.6 wt%, and NaCl concentration *ϕ* = 1 wt%) at different times and T = 37 °C. (**b**) The pattern growth at different stages. Labels I, II, and III indicate three stage of formation: coffee ring formation, crystals growth, and water drying, respectively. Label IV correspond to the final deposit after the complete evaporation process. The green arrows indicate the areas where the water drying is observed. (**c**) FOS (Mean, Standard Deviation, Integrated density, Skewness and Kurtosis) and (**d**) GLCM (Energy, Inertia, Correlation, IDM and Entropy) texture parameters extracted during the pattern formation. The black lines separate the different stages.

To determine the range of applicability of the FOS and GLCM statistic, we explore the patten formation from the evaporation of drops containing different relative protein concentration. A sequence of images of such process is shown in Fig. 4a,b. At the beginning of the drop evaporation, the contact line extends outwards to increase the area of contact between the droplet and the surface, see Fig. 4a. This occurs because the contact line obeys to a force balance given by Young’s equation: *σ*_*sv*_ = *σ*_*sl*_ + *σ*_*lv*_cos(*θ*), where *σ*_*sl*_, *σ*_*lv*_ and *σ*_*sv*_ are the solid-liquid, liquid-vapor, and solid-vapor surface tensions, and *θ* the contact angle. The extension process ends once the mechanical equilibrium of the contact line is reached.

The interaction phenomenon among proteins molecules in the droplets is as follow: as the protein molecule has a non-uniformly surface, with some hydrophobic regions and large zones of charged and polar groups (hydrophilic regions), the attraction between proteins emerge due to interactions of opposite charge and any exposed hydrophobic regions on their surface^48–51^. The addition of salt compounds in protein solutions will form a double layer (Stern and diffuse) of counter-ions on the surface of proteins. As a result, similar charged proteins experiment attractive and repulsive forces through the interaction of their electrical double layer^28,50,52^. However, by increasing salt concentration (an effect that occurs during the evaporation), the number of water binding by proteins will decrease to interact with salt ions. After that, since a high concentration of salt ions make proteins less soluble (salting-out effect), forcing the water molecules surrounding proteins to displace into the bulk solution; the structure and thickness of the double layer and hydration shell around proteins will be disturbed^26,53,54^. Thus the hydrophobic regions of proteins become exposed making available new points of attraction with neighbor protein molecules. Accordingly, proteins molecules will aggregate and precipitate^28,52^.

Figure 4b shows that the formation of the deposits takes place in three stages of aggregation of proteins. In the first one, the coffee ring emerges due to the capillary flows move radially outwards to compensate the loss of mass. Here, in the *coffee ring stage*, the evaporation of water molecules occurs mostly from the edge of the droplet. The proteins adsorbed in the surface go through a competitive exchange. Binary proteins mixtures follow a desorption/adsoption model in which proteins with major concentration and higher diffusion coefficients (smaller molecular weight) adsorb first. Later, these proteins will be displaced by proteins with lower concentrations, smaller diffusivities (larger molecular weight) and conformationally flexible if they have higher surface affinity^55–59^. This phenomenon is commonly referred to as the “Vroman effect”^60–63^ and promotes the selective distribution of different proteins on a surface. A different relative protein concentration generates different molecular distributions on a surface. We must remark, however, that nowadays not existing model that can fully explain this effect^64^. Since the molecular weight of lyzosyme is four-time lower than BSA, the evaporation of droplets of binary mixture protein solution as BSA/LYZ allow the Vroman effect. Therefore, the specific distribution of these proteins on a surface results in specific aggregation processes between ions and proteins. Processes that do not change sequentially with the relative protein concentration. Thereafter, in the *crystals growth stage* nucleation points appear that generate crystal growth from the central region of the deposit to the edge. Finally, in the *water drying stage*, the reminiscent water molecules placed among crystals quickly evaporate to form the complete structural characteristic of the deposits. We must remark, however, that there are only a little bit differences in the pixel intensity among deposits in the water drying stage and a deposit completely formed, see the area surrounding the interlocked crystal indicated by the green arrows in Fig. 4b.

The FOS and GLCM texture parameters capture the different stage of the formation process of deposits. The evolution of the FOS texture parameters extracted from a deposit formation process is plotted in Fig. 4c. The Mean, Standard Deviation, and Integrated Density parameters grow from the coffee ring stage to the crystals growth stage. Clearly, this occurs because the crystal formation increase the intensity pixel and the roughness on the deposit. Thereafter, in the water drying stage, these texture parameter reduces to reach a final value from the complete deposit after the evaporation process. On the other hand, the Skewness and Kurtosis reduces because the image deposit histogram presents a more symmetrical pattern right-tailed in the coffee ring stage than histograms extracted from the subsequent stages of deposit drying. Finally, we must remark that these parameters can not distinguish the water drying stage from the rest.

The evolution of the GLCM texture parameters is shown in Fig. 4d. The Energy, Correlation, and IDM parameters reduce because the deposit in the coffee ring stage show greater textural uniformity, similarity on gray-level regions, and local homogeneity than the deposit in subsequent stages. The Inertia and the Entropy increase during the deposit formation because the number of pixels in large contrast as well as heterogeneous regions in the deposit grow when more crystals are formed. Finally, we must remark that the FOS (mean, standard deviation, integrated density, skewness, and kurtosis) and GLCM (energy, inertia, correlation, IDM, and entropy) texture parameters capture the whole formation of the deposits, even if they are assembled by many complex aggregates.

## Discussion

The majority of investigators with interest in the structural characterization of deposits use empirical observations to identify shapes. For example, to the detection of different proteins, glycans, hepatotoxins and a malaria biomarker; the droplet evaporation method aim is to recognize the coffee ring on the deposits^65–68^. If such structure is identified, the presence of the molecule or the diagnostic of the pathology is positive; in contrast, the diagnostic is negative if the deposit shows a uniform coating. Unfortunately, salt is ubiquitous in biofluids and affect the formation of uniform coatings and the structure of the coffee rings. Nonetheless, in the context of diagnosis through the analysis of deposits, the complex structures made up of salts could serve as markers^10^.

Results show that the FOS and GLCM analysis can be a valuable tool to distinguish among deposits containing different concentrations of protein and salt. However, the image analysis has less consistent results to differentiate among deposits formed at high NaCl concentrations. This is probably because of the high number of crystals of different shapes which saturate the difference in the frequency and the intensity of the values of the grey levels of each pixel of the image. Indeed, these crystals diminish the influence of the large aggregates that appear covering small regions in the deposits formed at 1.0 wt%. Therefore, the asymptotic behavior of the curves of the parameters in Fig. 1 could reflect a threshold for the saturation of aggregates on protein deposits. On the other hand, the texture of the deposits does not change sequentially with the relative concentration of two structurally different proteins. This occurs because the change in relative concentrations of proteins modifies the complex proteins exchange on the surface, generating different distributions of proteins for the different relative concentrations. In other words, the different relative protein concentration modifies the dynamics of the Vroman effect and, as a consequence, the formation of salt aggregates.

Our results have demonstrated to be a powerful tool in characterize deposits formed in a wide range of relative humidity. The curves of the FOS and GLCM parameters in Fig. 3 show that the image analysis reveals a morphological phase transition of the aggregates inside of the deposits. They evolve from amorphous aggregates (at low RH) to branched arborescent patterns (at high RH). Furthermore, this technique can detect the different stages of aggregation that emerge due to the mass transport mechanisms, and intricate interaction among molecules, ions, and the substrate. We must remark that not all the variables recognize some small intermediate stages of aggregation. Indeed, only five parameters (standard deviation, integrated density, skewness, kurtosis, and the correlation) can distinguish these small aggregations stages. Nonetheless, all parameters reveal the morphological phase transition that arises from the evolution of a uniform deposit (which emerge due to protein deposition) to a complex deposit (which appear due to salt aggregation).

The pattern formation from the evaporation of droplets depends on some external parameters such as the temperature of the solid substrate, relative humidity of the evaporation environment (RH), and the air velocity. Furthermore, they depend on several internal conditions in the droplet such as the presence of surfactants, the physicochemical properties of the components of the solutions, and the size, the concentration, and chemical composition of the macromolecules. We have demonstrated the usefulness of FOS and GLCM texture analysis in the evaluation of deposits formed at different relative concentrations of proteins, NaCl, and relative humidity of the evaporation environment. Besides, the method can reveal the different aggregation stages during the creation of deposits patterns. This significant result suggests that the phenomenon of pattern formation in drying drops could be explored under this scheme regardless the control parameter that changes the transport mechanisms of mass and the aggregation processes in the interior of the droplet. Accordingly, this technique could satisfy the need of representing the complexity of a dried droplet of a biofluid of relevance used for diagnostic, on which a high number of physical-chemical variables contribute to its formation. Moreover, the method makes possible the evaluation of the surface texture of deposits directly from the digital image through histograms and two-dimensional arrays of co-occurrence. Therefore, this procedure is non-destructive, non-invasive, and rapid. We believe that this method could be used for rapid and constant monitoring of the pattern formation for medical testing in a laboratory. A general database could be created, allowing for people from all over the world to easily test their solutions.

On the other hand, the radial density, and normalized grayscale intensity profiles are standard quantities used to characterize and differentiate deposits. The first one measure the mass distribution as a function of the distance, from the center to the edge of the stain; while the second measures the distribution of mass on a narrow segment which covers the whole deposit. The accurate morphological characterization of deposits depends on the correct representation of the structural aspects of the deposits in the curves of these quantities. Unfortunately, these analyses are no very efficient. A small curve in the profiles of these quantities could be correlated with a large aggregate, a cluster of small aggregates, or a set of dispersed aggregates. Moreover, the normalized grayscale intensity profile captures only a small region of the structure of a deposit. The FOS and GLCM statistics capture the gray tone distribution of pixels intensity and the spatial arrangements of pixels intensities; which are two elements that emerge from the images of the complex structures inside of deposit. This analysis takes in the count all structures in the interior of the deposits regardless its shape, size, and number. The benefit of this image analysis compared to other ways of analyzing is that captures the whole structural aspects of deposits and collapse such information in a simple quantity that allows the direct comparison of the texture. This advantage could make possible to characterize and recognize a large number of complex biofluid deposits such as DNA/RNA, proteins, serum, blood, sperms cells, halophilic bacteria, among many other biofluids that containing relatively low salt concentrations.

In conclusion, we have present a texture analysis of deposits generated from the drying droplets of protein mixtures containing NaCl. Salt induce the formation of tip arrow-shapes, dendritic and semi-rosette patterns, needle-like and scalloped lines structures, and amorphous, star-like and prism-shaped salt crystals. The first-order statistics (FOS) and the gray level co-occurrence matrix (GLCM) analysis were carried out to capture the complex structural characteristic in such deposits. Interestingly, the parameters follow exponential functions that change as a function of the NaCl concentration because of reach saturation threshold in texture over protein deposits. This occurs because the formation of complex crystals changes the intensity pixels, the number of pixels in large contrast, and the number of heterogeneous regions in an image. Besides, the texture of the deposits does not change sequentially with the relative protein concentration because of Vroman effect induces changes the formation of salt aggregates. The combination of the FOS and GLCM parameters in the canonical discriminant analysis allows classifying deposits formed at different relative protein concentrations with an accuracy above 95%. Furthermore, a morphological change induced by the relative humidity (RH) appears in the aggregates located inside of the deposits. They evolve from amorphous aggregates (at low RH) to branched arborescent patterns (at high RH). Finally, FOS and GLCM statistics reveal a morphological phase transition that arises from a uniform deposit (formed by the deposition of proteins) to a complex deposit that arises due to aggregation of salt. This morphological phase transition emerges after three different stages of aggregation: coffee ring formation, the crystals growth, and water drying stage.

Overall, we have presented a methodology capable of quantifying changes in the pattern deposits. This finding suggests that the use of the first-order statistics and the gray level co-occurrence matrix statistics can be exploited to the assessment of pattern deposits associated to the biofluids of relevance, where the empirical interpretation of deposit images must be avoided.

## Methods

### Proteins preparation

High purity lysozyme (Sigma-Aldrich, L6876), bovine serum albumin (BSA) powders (Sigma-Aldrich, A2153) and sodium chloride (NaCl) were used to prepare stock solutions. These powders were dissolved in deionized water (Mili-Q, 18.2 *M*Ω⋅*cm*) to concentration of 2.00 wt% at 25 °C. The stock solutions, of proteins and NaCl, were diluted according to the desired relative protein concentration *ϕ*_*r*_ and NaCl concentration *ϕ*, respectively. Here, *ϕ*_*r*_ = A:B, where A and B stand for BSA and lysozyme protein molecules in the solution, respectively. The mixed protein solutions were prepared by mixing single protein solutions in varying ratios of BSA/LYZ, *ϕ*_*r*_ = 0:1, 1:51:1, 5:1, 1:0. Afterwards, we mixed BSA/LYZ and NaCl solutions in 1:1 volume ratio. The solutions were stored at 2 °C, thereafter thermalized to room temperature prior to deposition.

### Drop evaporation

The droplets of solutions were placed onto clean glasses slides using a micropipette, the volume of the drops was 2 *μl*. The droplets were evaporated under controlled ambient conditions: T = 25 and 37 °C; and relative humidity of from 13 to 90%. In order to explore the reproducibility, we carry out FOS and GLCM analysis over 24 deposits generates in two different experiments under the same conditions (twelve by sample). The analysis shows a high reliability because produces similar results for each sample.

The relative humidity was controlled by using the water activity effect ${a}_{w}=\frac{\rho }{{\rho }_{o}}$, where *ρ* is the water vapor pressure in the substance and *ρ*_*o*_ is the vapor pressure of pure water at the same temperature. Two containers with a saturated solution of NaCl with water activity *a*_*w*_ = 0.75 and distilled Water with *w* = 1 were placed in the box to reach RH = 70–90%, respectively. After that, to reduce the relative humidity to the desired values, different volumes of silica gel (Sigma-Aldrich, 13767) was used. The relative humidity values were measured with a high precision capacitive humidity controller (MH-1310A).

### Image acquisition

The deposits were observed after evaporation in ambient conditions using a microscope (Velab, VE-M4, 4x and 10x). The evaporation process was recorded at 30 fps with a digital camera (Nikon Digital, SLR Camera D3200). The resolution of the images was chosen to be 96 dpi, creating images with roughly 5470 × 4000 pixels. On the 8-bit images of is calculated the first-order statistics FOS (mean, standard deviation, integrated density, skewness and kurtosis) and the gray level co-occurrence matrix GLCM (energy, inertia, correlation, IDM and entropy) texture parameters. GLCM parameters were calculated for all four angles (0, 45, 90, 135) and were averaged in order not to take into account any geometric distributions on the surface of the material. The distance was 3.

### Texture analysis based on first-order statistics FOS

We used the histogram of the intensity values to extract some texture features through the mean value, standard deviation, integrated density, skewness, and kurtosis^69^. Mathematically, the histogram is defined as follows:

2 $$H(g)=\frac{{n}_{g}}{N};g=\mathrm{0,}\,\mathrm{1,}\,\mathrm{...,}\,{N}_{g}-1,$$

where *N* is the number of pixels in an image, *N*_*g*_ is the number of gray levels, and *n*_*g*_ is the number of pixels of value *g*. The following equations are used to calculate the FOS texture parameters:

*Mean*

3 $$\mu =\sum _{i=0}^{{N}_{g}-1}gH(g\mathrm{).}$$

*Standard deviation*

4 $$\sigma =\sqrt{\sum _{i=0}^{{N}_{g}-1}{(g-\mu )}^{2}H(g)}\mathrm{.}$$

*Integrated density*

5 $$I=\mu \cdot N.$$

*Skewness*

6 $${\mu }_{3}=\frac{\sum _{i=0}^{{N}_{g}-1}{(g-\mu )}^{3}H(g)}{{(\sigma )}^{3}}.$$

*Kurtosis*

7 $${\mu }_{4}=\frac{\sum _{i=0}^{{N}_{g}-1}{(g-\mu )}^{4}H(g)}{{(\sigma )}^{4}}-3.$$

Image intensity is usually represented by a mean value of pixels while the integrated density captures the total intensity of the pixels in an image. The standard variation is the intensity variation around the mean. Typically, high values of standard variation are related with a high roughness on the image surface. The skewness quantifies the asymmetry of a distribution concerning the mean value. In our images, lower skewness values appear in deposits where the aggregates are abundant. Finally, the kurtosis is a measure of the flatness of the histogram.

### Texture analysis based on gray level co-occurrence matrix (GLCM)

A gray level co-occurrence matrix (GLCM) is a matrix where the number of rows and columns is equal to the number of gray levels *N*_*g*_ in an image. Its analysis is based on the correlation among pixels in an image^70^. Mathematically, this information is captured by the matrix element *p*(*i*, *j*), which represent the probability values for changes between gray level *i* and *j* at a particular displacement distance (*d*) and angle (*ϕ*) on an image. This probability can be defined as:

8 $$p(i,j)=\frac{C(i,j)}{\sum _{i=0}^{{N}_{g}-1}\sum _{j=0}^{{N}_{g}-1}C(i,j)},$$

where *C*(*i*, *j*) is the number of occurrences of gray levels *i* and *j* within the window, at a particular (*d*, *ϕ*) pair. The denominator is the total number of gray level pairs (*i*, *j*) within the window and is bounded by an upper limit of *N*_*g*_*xN*_*g*_. The mean and the standard deviation for the columns and rows of the matrix, using the above equation, can be defined as follows:

9 $${u}_{x}=\sum _{i=0}^{{N}_{g}-1}\sum _{j=0}^{{N}_{g}-1}i\cdot p(i,j),\,{u}_{y}=\sum _{i=0}^{{N}_{g}-1}\sum _{j=0}^{{N}_{g}-1}j\cdot p(i,j),$$

10 $${\sigma }_{x}=\sum _{i\mathrm{=0}}^{{N}_{g}-1}\sum _{j\mathrm{=0}}^{{N}_{g}-1}{(i-{u}_{x})}^{2}\cdot p(i,j),\,{\sigma }_{y}=\sum _{i=0}^{{N}_{g}-1}\sum _{j=0}^{{N}_{g}-1}{(j-{u}_{y})}^{2}\cdot p(i,j),$$

where *u*_*x*_ and *u*_*y*_ are the mean for the columns and rows, respectively; and *σ*_*x*_ and *σ*_*y*_ represent the standard deviation for the columns and rows, respectively. Now, using these equations, we can define the texture parameter of GLCM as follows:

*Angular second moment* (*ASM*)

11 $$E=ASM=\sum _{i=0}^{{N}_{g}-1}\sum _{j=0}^{{N}_{g}-1}p{(i,j)}^{2}\mathrm{.}$$

*Moment of Inertia*

12 $$I=\sum _{i=0}^{{N}_{g}-1}\sum _{j=0}^{{N}_{g}-1}{(i-j)}^{2}p(i,j\mathrm{).}$$

*Correlation*

13 $$COR=U=\frac{\sum _{i=0}^{{N}_{g}-1}\sum _{j=0}^{{N}_{g}-1}(ij)\cdot p(i,j)-{u}_{x}{u}_{y}}{{\sigma }_{x}{\sigma }_{y}}.$$

*Inverse difference moment (IDM)*

14 $$IDM=\sum _{i=0}^{{N}_{g}-1}\sum _{j=0}^{{N}_{g}-1}\frac{1}{1+{(i-j)}^{2}}p(i,j\mathrm{).}$$

*Entropy*

15 $$H=-\,\sum _{i=0}^{{N}_{g}-1}\sum _{j=0}^{{N}_{g}-1}p(i,j)log(p(i,j\mathrm{)).}$$

Angular second moment (also known as energy) is a measure of global homogeneity of an image. Its quantity captures the degree of gray uniformity and texture coarseness. Higher (lower) energy values indicate textural uniformity (heterogeneity). The inertia captures the roughness and complexity of texture feature presented in an image. The more pixels in high contrast, the more significant moment of inertia. Correlation captures the degree of similarity between GLCM elements in a row or column direction. Higher (lower) correlation values indicate similar (different) gray-level regions. Inverse difference moment IDM captures the local homogeneity of an image. Higher IDM values indicate local homogeneity, while lower IDM is characteristic of inhomogeneous images. Entropy captures the randomness of the image texture. Higher (lower) entropy values indicate large (small) heterogeneous regions in an image.

## Electronic supplementary material

Supplementary information
