# Supplementary material for: Texture analysis of protein deposits produced by droplet evaporation
Source: Sci Rep. 2018 Jun 25;8:9580. doi: 10.1038/s41598-018-27959-0 (PMC6018122; doi:10.1038/s41598-018-27959-0)
Supplement: Supplementary file 1 — Supplementary information [file 41598_2018_27959_MOESM1_ESM.pdf]

Texture analysis of protein deposits produced by droplet evaporation

Yojana J.P. Carreón, M. Rios-Ramirez, R. E. Moctezuma, and Jorge González-Gutiérrez
Contact: drjorgegltzgtz@gmail.com

Fig. 1s shows protein deposits formed at relative protein concentration ϕ_r =0:1, 1:5, 1:1, 5:1, 1:0, protein contraction ϕ_p =1.6 wt%, and NaCl concentration ϕ = 0.5wt%. Fig. 2s shows the scatter diagram of the first two discriminant functions from the GLCM parameters. Table 1 shows the unstandardized canonical coefficients corresponding to the canonical discriminant functions obtained from the discriminant analysis using GLCM. Table 2 shows small Wilks lambda values related to the above discriminant functions. Table 3 shows the accuracy in the classification of the groups of deposits.

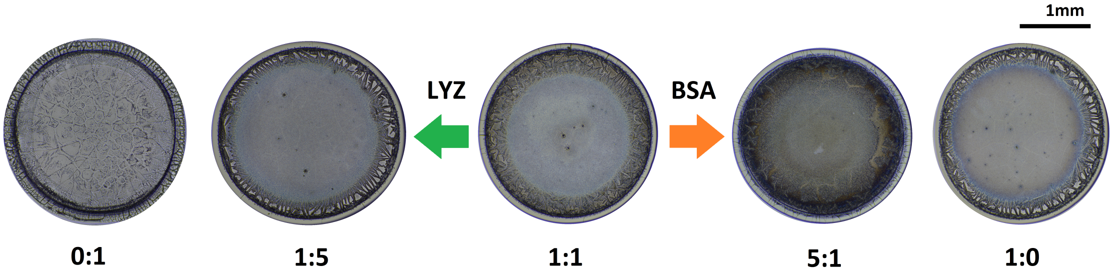

Supplementary Fig.1s Protein mixtures deposits formed at different relative concentration. Deposits containing proteins mixture of BSA-LYZ. Relative protein concentration ϕ_r =0:1, 1:5, 1:1, 5:1, 1:0, protein contraction ϕ_p =1.6 wt%, and NaCl concentration $\phi = 0.5$wt% (black squares and red circles, respectively). The droplet evaporation was carry out on a solid surface at T= 37 °C.

	FOS + GLCM	FOS + GLCM	FOS	GLCM	GLCM	GLCM	GLCM
	Cano. Var.1	Cano. Var.2	Cano. Var.1	Cano. Var.1	Cano. Var.2	Cano. Var.3	Cano. Var.4
Constant	-16.385	-9.989	-18.685	-196.986	146.595	-98.24	14.531
B	-5.883x10^{-9}	5.979x10^{-9}	6.405x10^{-14}	-	-	-	-
C	-1.849x10^{-2}	1.880x10^{-2}2	-6.652x10^{-15}	-	-	-	-
D	-1.211x10^{-9}	1.231x10^{-9}	9.215x10^{-8}	-	-	-	-
E	1.376x10^{-6}	-1.399x10^{-6}	-2.226x10^{-15}	-	-	-	-
F	-3.208x10^{-5}	3.260x10^{-5}	-2.379x10^{-15}	-	-	-	-
G	1.570x10^{-3}	-1.600x10^{-3}	-	2302.252	-869.549	-44.409	-436.882
H	-4.143x10^{-4}	4.211x10^{-4}	-	6.830x10^{-3}	3.137x10^{-2}	-1.060x10^{-2}	2.078x10^{-2}
I	1.0605x10^{-7}	2.360x10^{-8}	-	24773.133	-9359.119	-484.606	-4702.301
J	8.565x10^{-6}	-8.704x10^{-6}	-	128.098	-4.674	120.72673	-0.988
K	1.110x10^{-4}	-1.128x10^{-4}	-	17.418	-17.449	8.777	-1.987

TABLE I. The unstandardized canonical coefficients. The discriminant analysis give two, fourth, and one canonical discriminant functions for from the complete sets of FOS and GLCM parameters, the FOS parameters, and the GLCM parameters, respectively. The coefficients serves to find out the best coefficient estimation to maximize the difference in mean discriminant score between groups. Cano. var. are the canonical variables of the canonical discriminant functions.

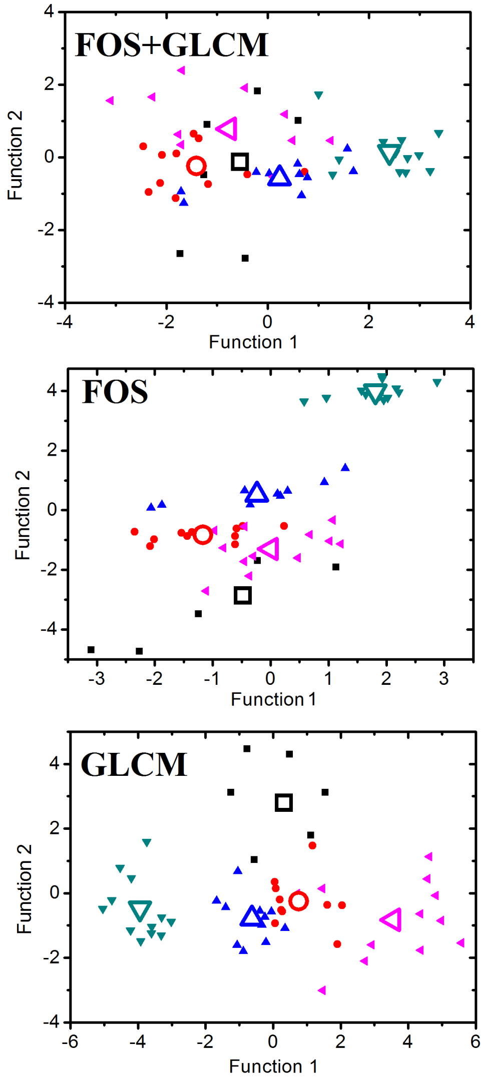

Supplementary Fig.2s The scatter diagram for the sample sites with the first two discriminant functions. The clearer the observations are grouping to, the better the discriminant model is. The deposit groups are indicate as follows: 0:1 black squares, 1:5 red circles, 1:1 blue triangles, 5:1 green triangles; and 1:0 magenta triangles. The big symbols are the corresponding group centroid.

	Wilks' Lambda	Chi-square	significance
FOS + GLCM	0.274	69.188	0.000
FOS + GLCM	0.810	11.241	0.010
FOS	0.474	40.261	0.000
GLCM	0.024	194.173	0.000
GLCM	0.188	87.643	0.001
GLCM	0.534	32.869	0.002
GLCM	0.919	4.419	0.035

TABLE II. Wilks lambda test for each model.

Error rate						
Group	0:1	1:5	1:1	5:1	1:0	Total
FOS + GLCM	0%	0%	0%	0%	0%	0%
FOS	0%	0%	0%	0%	25%	5%
GLCM	0%	8.33%	8.33%	0%	16.6%	6.67%

TABLE III. The Error Rate. The table lists the prior probability of each groups and the rate for misclassification.
